# Comparison of haplo-SCT and chemotherapy for young adults with standard-risk Ph-negative acute lymphoblastic leukemia in CR1

**DOI:** 10.1186/s13045-020-00879-1

**Published:** 2020-05-15

**Authors:** Meng Lv, Qian Jiang, Dao-Bin Zhou, Yu Hu, Dai-Hong Liu, De-Pei Wu, Jing-Bo Wang, Hao Jiang, Jing Wang, Ying-jun Chang, Yu Wang, Xiao-Hui Zhang, Lan-Ping Xu, Kai-Yan Liu, Xiao-Jun Huang

**Affiliations:** 1 Peking University People’s Hospital, Peking University Institute of Hematology, Beijing Key Laboratory of Hematopoietic Stem Cell Transplantation, National Clinical Research Center for Hematologic Disease, Beijing, China; 2 Peking Union Hospital, Chinese Academy of Medical Sciences and Peking Union Medical College, Beijing, China; 3 Wuhan Union Hospital, Tongji Medical College, Huazhong University of Science and Technology, Wuhan, China; 4 General Hospital of PLA (People’s Liberation Army of China), Beijing, China; 5 The First Affiliated Hospital of Soochow University, National Clinical Research Center for Hematologic Disease, Suzhou, China; 6 Peking University Aerospace Center Hospital, Beijing, China; 7 Peking-Tsinghua Center for Life Sciences, Beijing, China; 8 Research Unit of Key Technique for Diagnosis and Treatments of Hematologic Malignancies (2019RU029), Chinese Academy of Medical Sciences, Beijing, China

**Keywords:** Haplo-SCT, Adult chemotherapy, Ph-negative acute lymphoblastic leukemia

## Abstract

**Abstract:**

Human leukocyte antigen (HLA) haploidentical stem cell transplantation (haplo-SCT) as a postremission treatment for standard risk Philadelphia chromosome-negative acute lymphoblastic leukemia (SR Ph-ALL) in the first complete remission (CR1) has not been defined. In this multicenter, phase 3 study (NCT02042690), of the 131 consecutive Ph-ALL young adult patients (YA, aged 18–39 years) without high-risk features who achieved CR1, 114 patients without HLA-matched donors received consolidation with an adult chemotherapy regimen (*n* = 55) or haplo-SCT (*n* = 59). In the landmark analysis, haplo-SCT resulted in a lower 2-year cumulative incidence of relapse (CIR, 12.8% vs 46.7%, *P* = 0.0017) and superior 2-year leukemia-free survival (LFS, 80.9% vs 51.1%, *P* = 0.0116) and 2-year overall survival (OS, 91.2% vs 75.7 [64.8–93.2] %, *P* = 0.0408) than chemotherapy. In the time-dependent multivariate analysis with propensity score adjustment, postremission treatment (haplo-SCT vs chemotherapy) was an independent risk factor for the CIR (HR 0.195, 95% CI 0.076–0.499, *P* = 0.001), LFS (HR 0.297, 95% CI 0.131–0.675, *P* = 0.003), and OS (HR 0.346, 95% CI 0.140–0.853, *P* = 0.011). In all subgroups, CIR was lower in haplo-SCT. Myeloablative haplo-SCT with ATG+G-CSF might be one of the preferred therapies for YA patients with standard-risk Ph-ALL.

**Trial registration:**

ClinicalTrials.gov. Registered on 23 January 2014, https://clinicaltrials.gov/ct2/show/NCT02042690

To the Editor:

Philadelphia chromosome-negative acute lymphoblastic leukemia (Ph-ALL) is categorized as high risk (HR) with risk factors such as advanced age, elevated WBC count, and high-risk cytogenetic abnormalities. The remaining older adolescents and young adults (AYA, aged 15–39 years) without risk factors are AYA with standard-risk (SR) Ph-ALL and represent a group with lower cumulative incidence of relapse and better overall survival. Allogeneic hematopoietic stem cell transplantation (allo-SCT), especially from human leukocyte antigen (HLA)-matched sibling donors (MSDs) or matched unrelated donors (MUDs), is one of the preferred options over chemotherapy in the consolidation treatment of Ph-ALL [1, 2]. However, the shortage of MSDs and limited availability of MUDs prevents large populations from benefiting from allo-SCT [3].

Recently, unmanipulated haploidentical SCT (haplo-SCT) using pretransplant ATG and granulocyte colony-stimulating factor (G-CSF)-stimulated grafts (ATG+G-CSF) or posttransplant cyclophosphamide (PT-CY) protocol was confirmed equivalent to HLA-matched SCT in ALL [4–6]. However, as prospective data is absent, it is unknown whether AYA SR Ph-ALL patients should pursue haplo-SCT instead of consolidation chemotherapy in the absence of MSDs and MUDs [7, 8]. This multicenter prospective clinical trial was registered at https://clinicaltrials.gov as NCT02042690 (Suppl Method).

In total, 131 consecutive Ph-ALL young adult patients (YA, aged 18–39 years) without high-risk features who achieved CR1 were enrolled with a median follow-up of 32 months (Figure S1, Table S1). haplo-SCT was superior to chemotherapy in terms of lower CIR and improved LFS and OS in total enrolled CR1 patients without landmark (Figure S2); haplo-SCT was also associated with lower CIR and improved LFS in the subgroup of patients who took only 1 cycle to achieve CR and been MRD negative after Con-1 (Figure S3).

Dynamic landmark suggested haplo-SCT was associated with lower CIR and improved LFS and OS compared with chemotherapy between 0 and 12 months post-CR1(Figure S4). Then, 6 months was chosen as the fixed landmark point, relapse or NRM before 6 months post-CR1 (*n* = 15) was excluded, those undergoing SCT after the landmark were included in the chemotherapy group, and the remaining patients (*n* = 99) were divided into the haplo-SCT group (*n* = 49) and chemotherapy group (*n* = 50) (Table S2). In landmark analysis, CIR (2-year CIR 12.8%, 95% CI 3.2–22.4 vs 46.7%, 95% CI 30.5–52.9%; *P* = 0.0017), LFS (2-year LFS 80.9%, 95% CI 66.4–89.6 vs 51.1%, 95% CI 34.2–65.6%; *P* = 0.0116), and OS (2-year OS 91.2%, 95% CI 78.2–96.6% vs 75.7%, 95% CI 64.8–93.2%; *P* = 0.0408) continued to be better in the haplo-SCT group than in the chemotherapy group (Fig. 1a, c, d), while NRM was comparable (Fig. 1b).

**Fig. 1:**
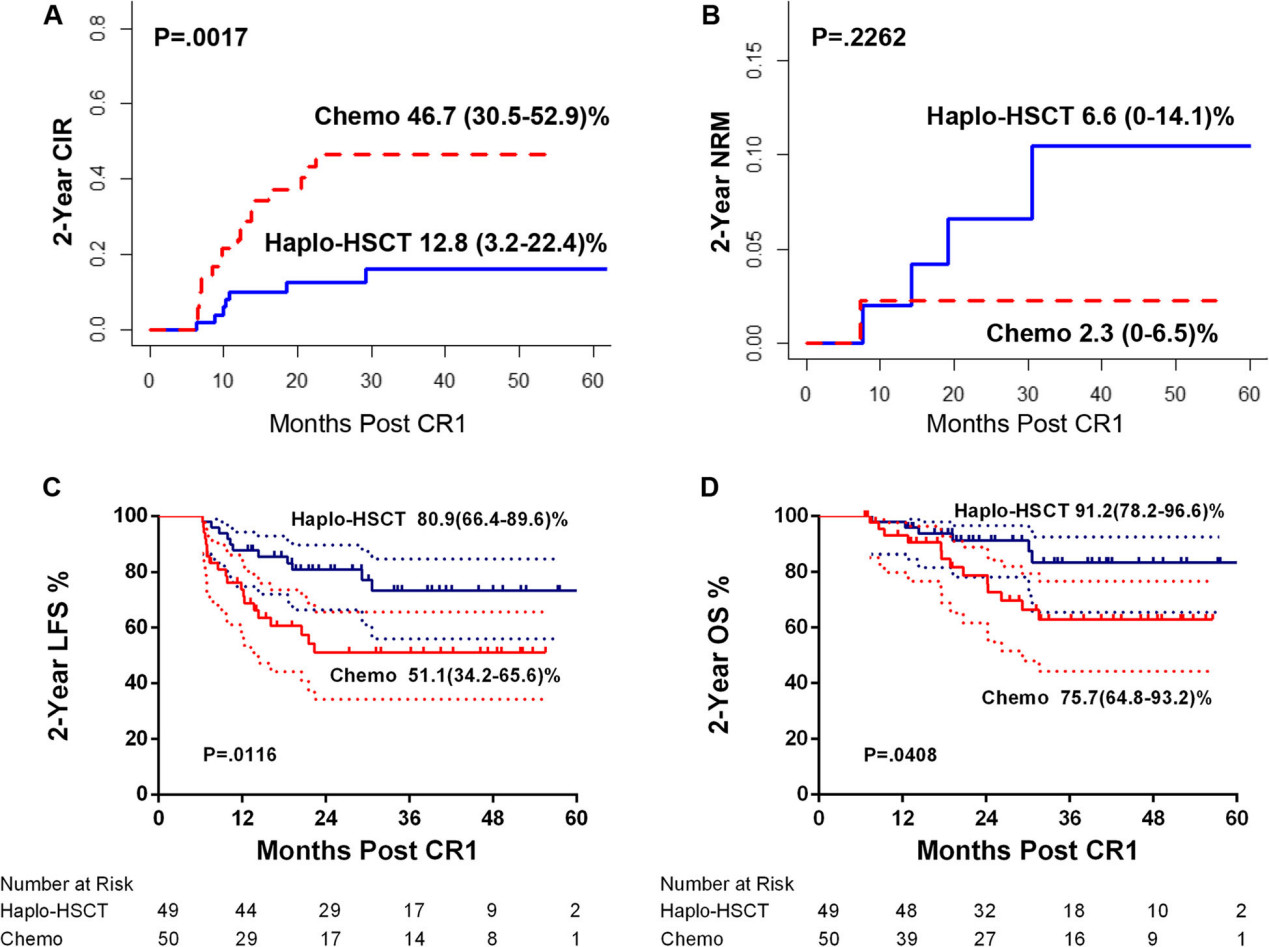
Comparison between chemotherapy and haplo-SCT with landmark. **a** Cumulative incidence of relapse (CIR). **b** Non-relapse mortality (NRM). **c** Leukemia-free survival (LFS). **d** Overall survival (OS)

Cox PH regression model was constructed considering the time of haplo-SCT as a time-dependent exposure based on PH test (Table S3). Univariate analysis for CIR, NRM, LFS, and OS was listed in Table S4. Both crude- and PS-adjusted multivariate analyses suggested haplo-SCT was associated with lower CIR (PS-adjusted HR 0.195, 95% CI 0.076–0.499, *P* = 0.001) and improved LFS (PS-adjusted HR 0.297, 95% CI 0.131–0.675, *P* = 0.003) and OS (HR 0.346, 95% CI 0.140–0.853, *P* = 0.011) compared with chemotherapy. Con1 FCM MRD (+ vs −) was an independent risk factor for CIR (PS-adjusted HR 3.609, 95% CI 1.562–8.340, *P* = 0.006) and LFS (PS-adjusted HR 2.825, 95% CI 1.298–6.152, *P* = 0.009). Diagnosis (T vs B; HR 2.564, 95% CI 1.361–4.823, *P* = 0.014) was an independent risk factor for OS. No independent risk factors identified for NRM. When stratified by Con-1 MRD and diagnosis, haplo-SCT decreased CIR in all subgroups (Con-1 MRD+ vs MRD−, B-ALL vs T-ALL) while improved LFS and OS only in the Con-1 MRD+ and B-ALL subgroups but not in the Con-1 MRD− and T-ALL subgroups (Table S5, Fig. 2).

**Fig. 2:**
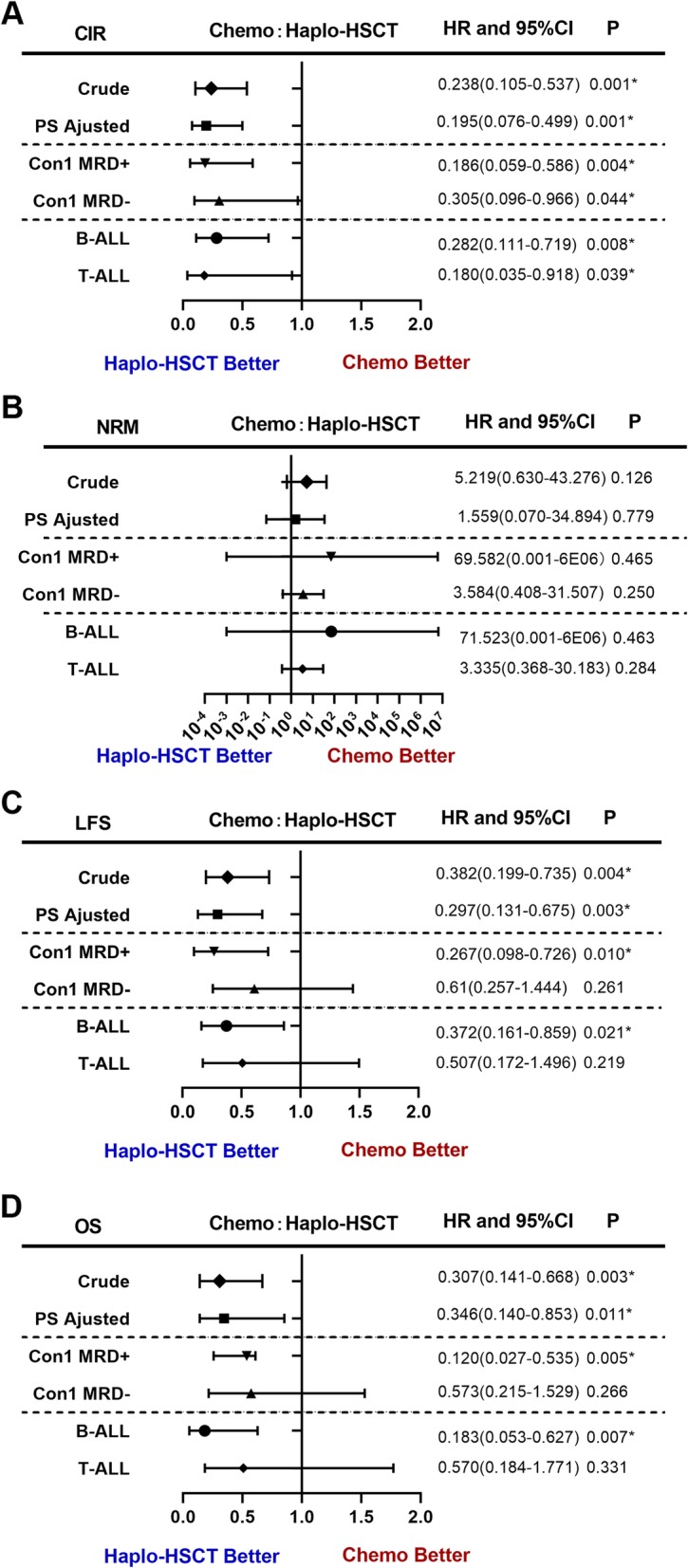
Forest plot of time-dependent multivariable Cox regression model. **a** Cumulative incidence of relapse (CIR). **b** Non-relapse mortality (NRM). **c** Leukemia-free survival (LFS). **d** Overall survival (OS)

Currently, haplo-SCT is only an optional rather than a preferred choice for postremission therapy compared with MSD or 10/10 MUD-SCT MSD-SCT is the preferred treatment for ALL, and MUD is also acceptable in most countries [7]. This study presents the first prospective assessment related to the controversial issue whether YA patients with SR ALL benefit more from haplo-SCT than adult chemotherapy regimen. The advantages of a low CIR and an acceptable NRM resulted in promising results of haplo-SCT in the present study, which were comparable to those in previous reports (5-year LFS 68.7%, OS 70.1%) [5, 8]. As NRM of haplo-SCT has generally improved with either the PT-CY (7 to 23%) or ATG+G-CSF protocol (11–13%) compared with early procedures [4, 9, 10], NRM might no longer be a limiting factor of receiving haplo-SCT, especially in experienced centers. haplo-SCT was associated with lower CIR in both the Con-1 MRD +/− subgroups in the current study; meanwhile, cautions must be taken as CIR of non-SCT cohort might be higher compared with previous reports (46–49%) [1, 2]. More recently, some studies suggested pediatric-inspired regimens might further decrease the CIR to 12–33% and result encouraging survival (3–5 years LFS 59–73%, OS 60–79%) compared with adult regimen [11], while some reported similar outcomes [12]. Currently, guidelines tried to recommend the regimens both by adult and pediatric settings as adult regimens were still widely used, especially in developing countries [13, 14]. In addition, blinatumomab, which might further decrease CIR in MRD+ALL [15], was not available in the current study. Therefore, it remained to be addressed the role of haplo-SCT in the era of pediatric-inspired regimens and blinatumomab in the future.

The present study might be one of the best available evidence to compare haplo-SCT and adult chemotherapy for YA SR Ph-ALL in CR1. Cautions must be taken in interpreting these results due to non-randomized design and a relatively small group of patients. haplo-SCT might become one of the preferred therapies for YA patients with SR Ph-ALL in the absence of MSD or MUD-SCT.

## Supplementary information

**Additional file 1:.** Supplementary figures.

**Additional file 2:.** Supplementary tables.

**Additional file 3:.** Supplementary methods.

## Data Availability

All data generated or analyzed during this study are included in this published article and its supplementary information files.

## Abbreviations

allo-SCT: Allogeneic hematopoietic stem cell transplantation
AYA: Adolescents and young adults
CIR: Cumulative incidence of relapse
G-CSF: Granulocyte colony-stimulating factor
GRFS: GVHD-free, relapse-free survival rates
GVHD: Graft-versus-host disease
haplo-SCT: Haploidentical SCT
HLA: Human leukocyte antigen
HR: High risk
LFS: Leukemia-free survival
MRD: Minimal residual disease
MSDs: Matched sibling donors
MUDs: Matched unrelated donors
NRM: Non-relapse mortality
OS: Overall survival
Ph-ALL: Philadelphia chromosome-negative acute lymphoblastic leukemia
PS: Propensity score
PT-CY: Posttransplant cyclophosphamide
SR: Standard-risk

## References

[1] Goldstone AH, Richards SM, Lazarus HM, Tallman MS, Buck G, Fielding AK (2008). In adults with standard-risk acute lymphoblastic leukemia, the greatest benefit is achieved from a matched sibling allogeneic transplantation in first complete remission, and an autologous transplantation is less effective than conventional consolidation/maintenance chemotherapy in all patients: final results of the international ALL trial (MRC UKALL XII/ECOG E2993). Blood.

[2] Cornelissen JJ, van der Holt B, Verhoef GE, van’t Veer MB, van Oers MH, Schouten HC (2009). Myeloablative allogeneic versus autologous stem cell transplantation in adult patients with acute lymphoblastic leukemia in first remission: a prospective sibling donor versus no-donor comparison. Blood.

[3] Aljurf M, Weisdorf D, Alfraih F, Szer J, Muller C, Confer D (2019). Worldwide network for Blood & Marrow Transplantation (WBMT) special article, challenges facing emerging alternate donor registries. Bone Marrow Transplant.

[4] Wang Y, Liu QF, Xu LP, Liu KY, Zhang XH, Ma X (2016). Haploidentical versus matched-sibling transplant in adults with Philadelphia-negative high-risk acute lymphoblastic leukemia: a biologically phase III randomized study. Clin Cancer Res.

[5] Han LJ, Wang Y, Fan ZP, Huang F, Zhou J, Fu YW (2017). Haploidentical transplantation compared with matched sibling and unrelated donor transplantation for adults with standard-risk acute lymphoblastic leukaemia in first complete remission. Br J Haematol.

[6] Shem-Tov N, Peczynski C, Labopin M, Itala-Remes M, Blaise D, Labussiere-Wallet H (2020). Haploidentical vs. unrelated allogeneic stem cell transplantation for acute lymphoblastic leukemia in first complete remission: on behalf of the ALWP of the EBMT. Leukemia.

[7] Giebel S, Marks DI, Boissel N, Baron F, Chiaretti S, Ciceri F (2019). Hematopoietic stem cell transplantation for adults with Philadelphia chromosome-negative acute lymphoblastic leukemia in first remission: a position statement of the European working Group for Adult Acute Lymphoblastic Leukemia (EWALL) and the acute leukemia working Party of the European Society for blood and marrow transplantation (EBMT). Bone Marrow Transplant.

[8] Yan CH, Jiang Q, Wang J, Xu LP, Liu DH, Jiang H (2014). Superior survival of unmanipulated haploidentical hematopoietic stem cell transplantation compared with chemotherapy alone used as post-remission therapy in adults with standard-risk acute lymphoblastic leukemia in first complete remission. Biol Blood Marrow Transplant.

[9] Bashey A, Zhang M-J, McCurdy SR, St. Martin A, Argall T, Anasetti C (2017). Mobilized peripheral blood stem cells versus unstimulated bone marrow as a graft source for T-cell–replete haploidentical donor transplantation using post-transplant cyclophosphamide. J Clin Oncol.

[10] Sestili S, Labopin M, Ruggeri A, Velardi A, Ciceri F, Maertens J (2018). T-cell-depleted haploidentical stem cell transplantation results improve with time in adults with acute leukemia: a study from the acute leukemia working Party of the European Society of blood and marrow transplantation (EBMT). Cancer.

[11] Siegel SE, Stock W, Johnson RH, Advani A, Muffly L, Douer D (2018). Pediatric-inspired treatment regimens for adolescents and young adults with Philadelphia chromosome-negative acute lymphoblastic leukemia: a review. JAMA Oncol.

[12] Rytting ME, Jabbour EJ, Jorgensen JL, Ravandi F, Franklin AR, Kadia TM (2016). Final results of a single institution experience with a pediatric-based regimen, the augmented Berlin-Frankfurt-Munster, in adolescents and young adults with acute lymphoblastic leukemia, and comparison to the hyper-CVAD regimen. Am J Hematol.

[13] NCCN guideline: Acute lymphoblastic leukemia. Version2.2019; http://www.nccn.org.

[14] NCCN guideline: Pediatric acute lymphoblastic leukemia. Version1.2020; http://www.nccn.org.

[15] Curran E, Stock W (2019). Taking a “BiTE out of ALL”: blinatumomab approval for MRD-positive ALL. Blood.

