# Supplementary material for: Comparison of haplo-SCT and chemotherapy for young adults with standard-risk Ph-negative acute lymphoblastic leukemia in CR1
Source: J Hematol Oncol. 2020 May 15;13:52. doi: 10.1186/s13045-020-00879-1 (PMC7227076; doi:10.1186/s13045-020-00879-1)
Supplement: Supplementary file 1 — Additional file 1:. Supplementary figures. [file 13045_2020_879_MOESM1_ESM.docx]

**Figure S1 Figure 1. Overview of consecutive patients included in the present trial.**

ALL, acute lymphoblastic leukemia; SR, standard risk; CR, complete remission; Chemo, chemotherapy; SCT, hematopoietic stem-cell transplantation; Haplo, haploidentical related donor; MSD, matched sibling donor; MUD, matched unrelated donor.

**
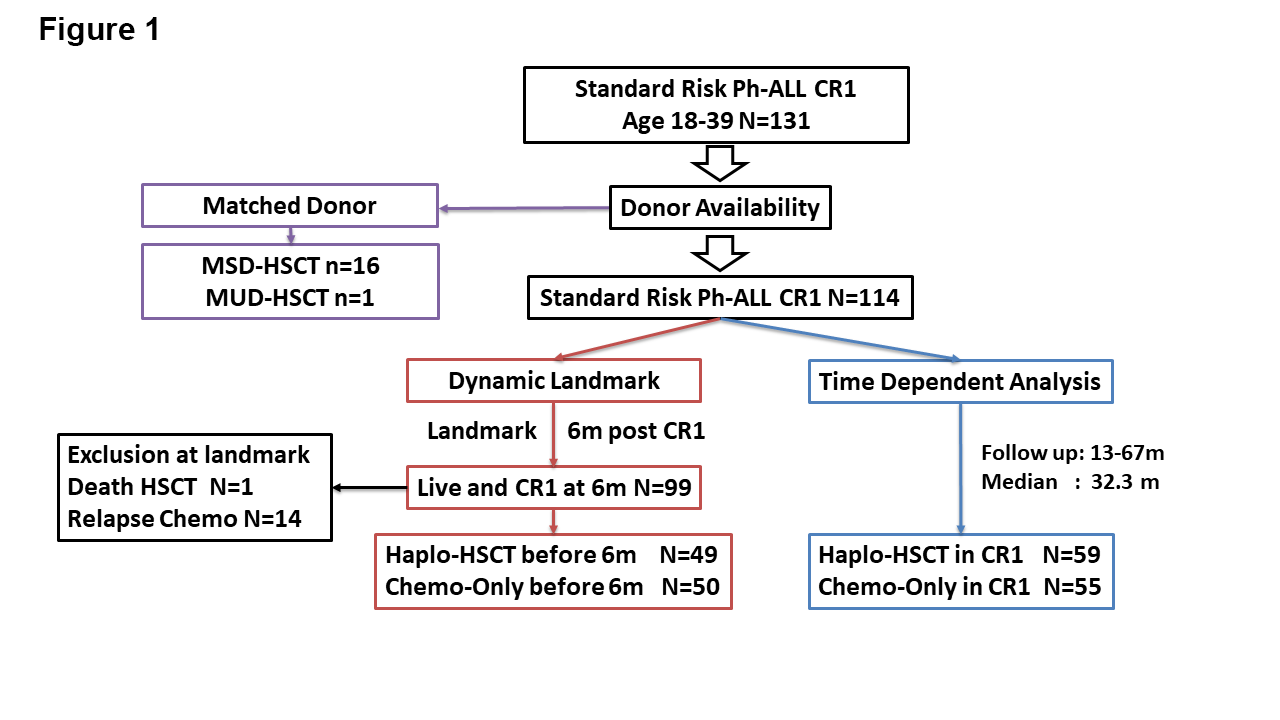
**

**Figure S2. Outcomes of total enrolled CR1 patients and comparison between chemotherapy and haplo-SCT without landmark.**

(A) Cumulative incidence of relapse (CIR) in total enrolled CR1 patients; of the 131 patients in CR1, 46 experienced relapse, which corresponds to a 2-year CIR of 36.3% (symmetric 95% CI, 27.7-44.9); 24 of the 33 patients relapsed before SCT led to a second CR (CR2; 72.7% of whom relapsed), and 12 patients underwent allo-SCT. In addition, 13 cases of relapse occurred post allo-SCT (8 cases after haplo-SCT, 5 after MSD-SCT), and 6 patients achieved durable remission. (B)CIR was significantly higher in the chemotherapy group (2-year 62.7%, symmetric 95% CI 49.3-76.1) than in the haplo-SCT group (2-year 12.7%, symmetric 95% CI 3.9-21.5, P<.0001).；(C) Nonrelapse mortality (NRM) in total enrolled CR1 patients; there was 1 NRM case in the chemotherapy group, and there were 9 cases in the SCT group (7 after haplo-SCT, 2 after MSD-SCT), which corresponds to a 2-year CIR of 7.2% (symmetric 95% CI, 2.3-12.1), (D) NRM was worse in the haplo-SCT group (2-year 9.5%, symmetric 95% CI 1.5-17.5) than in the chemotherapy group (2-year 2.0%, symmetric 95% CI 0-5.9; P=0.0269); (E) The 2-year Leukemia-free survival (LFS) of all patients in CR1 were 57.9% (asymmetric 95% CI, 48.6-66.1), (F) LFS was significantly lower in the chemotherapy group(2-year LFS 36.4%, asymmetric 95% CI 23.5-39.3) than in the haplo-SCT group(78.6%, asymmetric 95% CI 69.9-87.0; P<0.0001) without landmark；(G) The 2-year Overall survival (OS) of all patients in CR1 were 73.2% (asymmetric 95% CI, 64.1-80.3); (H)OS was significantly lower in the chemotherapy group(63.3%, 95% CI 46.2-74.9)than in the haplo-SCT group (88.8%, asymmetric 95% CI 76.6-94.8; P=0.0018) without landmark.

***
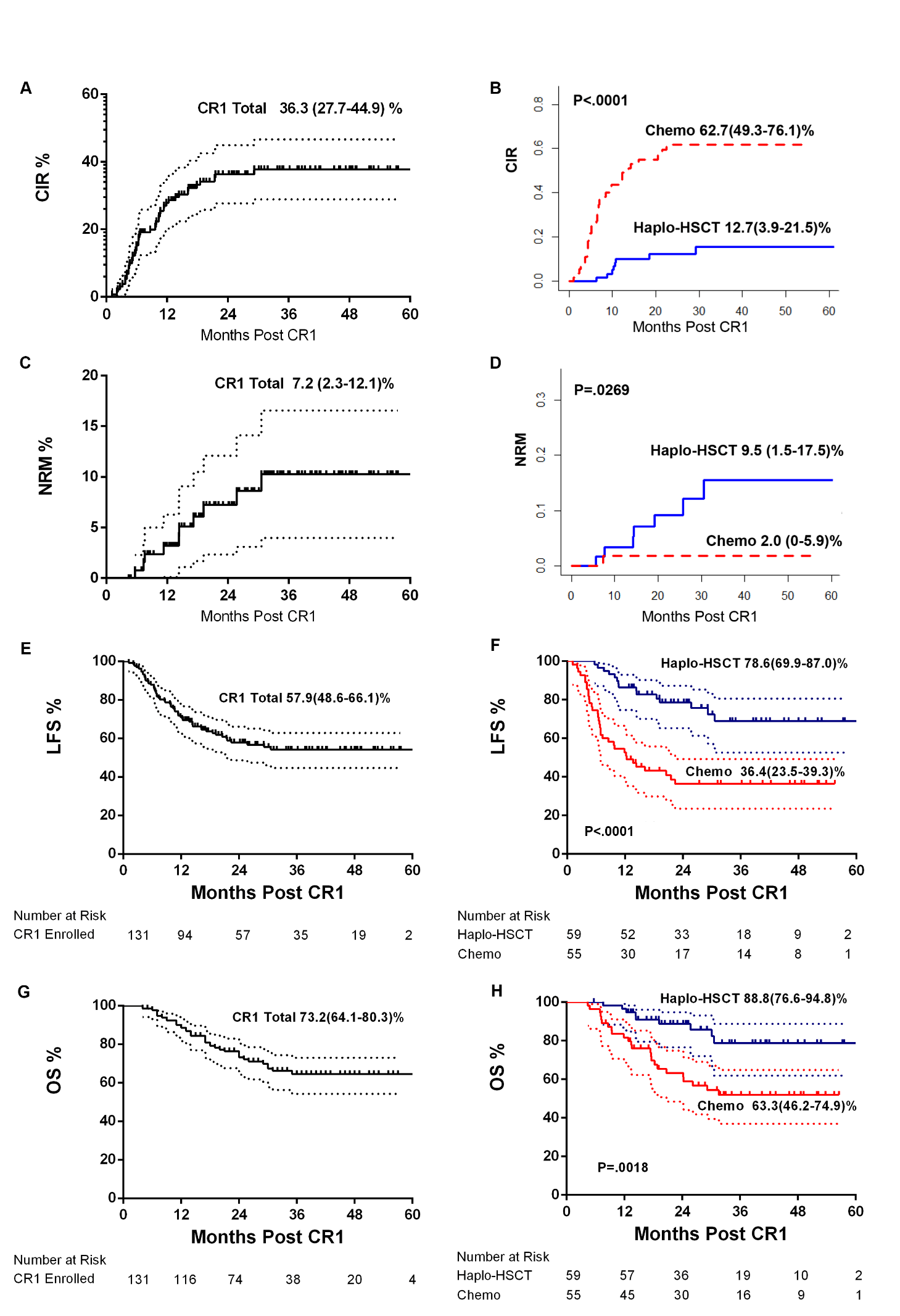
***

**Figure S3. Outcomes of patients who** **took only 1 cycle to achieve CR and with negative MRD after Con-1 without landmark.**

Of the total 131 patients in CR1, 59 patients took only 1 cycle to CR1 and got negative MRD after Con-1, in which 29 patients only received chemotherapy while 30 patients received haplo-SCT in CR1.

(A) CIR was significantly higher in the chemotherapy group (2-year 42.8%, symmetric 95% CI 33.2-52.4%) than in the haplo-SCT group (2-year 14.1%, symmetric 95% CI 7.4-20.8, P=0.0050)； (B) NRM was comparable in the haplo-SCT group and chemotherapy group (haplo-SCT: symmetric 2-year 12.8%, 95% CI 7.4-18.2%; chemotherapy: 2-year 3.4%, 95% CI 2.2-4.6; P=0.1490); (C) LFS was significantly lower in the chemotherapy group than in the haplo-SCT group without landmark (2-year LFS 53.7%, asymmetric 95% CI 33.6-66.9 vs. 85.5%, 95% CI 65.7-94.3; P=0.0481； (D)OS was comparable in the chemotherapy group and in the haplo-SCT group (74.9%, asymmetric 95% CI 54.2-87.3 vs.92.9%, 95% CI 74.6-98.1; P=0.1549).

**
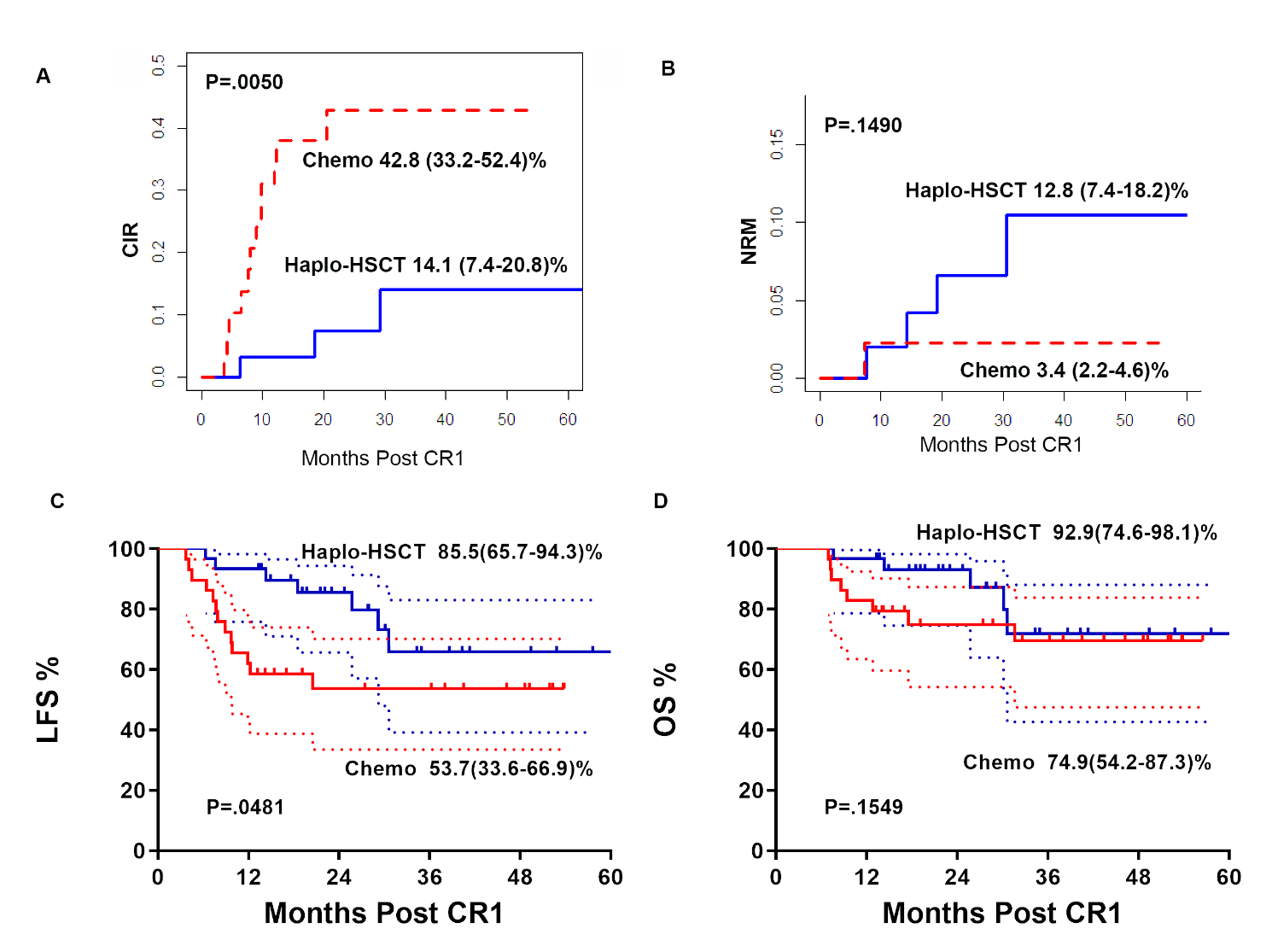
**

**Figure S4** **Dynamic landmark comparing Haplo-HSCT with chemotherapy**

Haplo-SCT was compared with chemotherapy in dynamic landmark analysis between 0 and 12 months post-CR1: **(**A) Haplo-SCT was associated with lower cumulative incidence of relapse (CIR) (B=-1.532, Exp(B)/HR=0.216, P=0.002); (B) Haplo-SCT was associated with improved Leukemia-free survival (LFS) (B=-0.816, Exp(B)/HR =0.442, P=0.014); (C) Haplo-SCT was associated with improved Overall survival (OS) (B=-0.571, Exp(B)/HR =0.565, P=0.047).

**
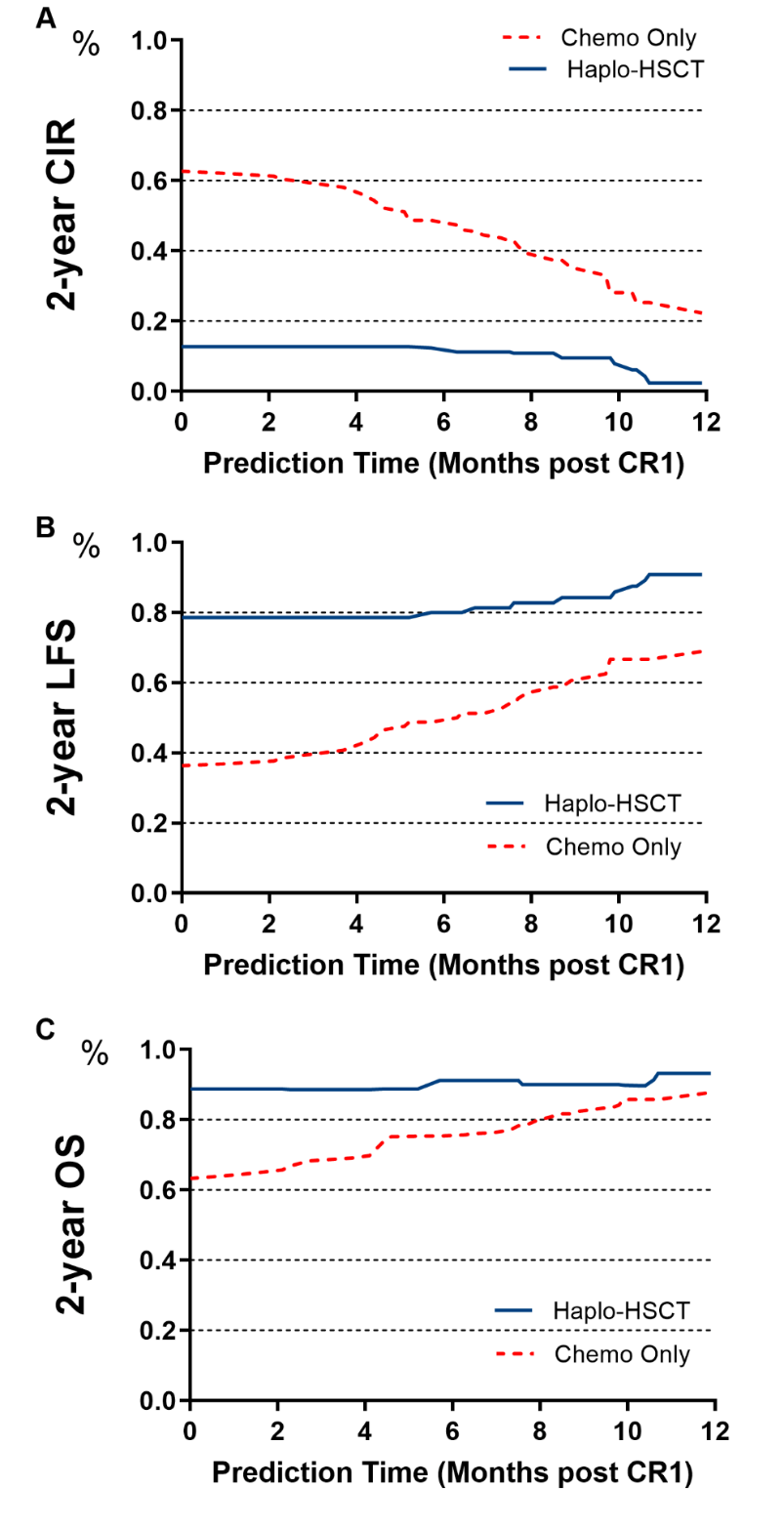
**
