# Supplementary material for: Comparison of haplo-SCT and chemotherapy for young adults with standard-risk Ph-negative acute lymphoblastic leukemia in CR1
Source: J Hematol Oncol. 2020 May 15;13:52. doi: 10.1186/s13045-020-00879-1 (PMC7227076; doi:10.1186/s13045-020-00879-1)
Supplement: Supplementary file 2 — Additional file 2:. Supplementary tables. [file 13045_2020_879_MOESM2_ESM.docx]

**Table S1 Patient characteristics of haplo-SCT and chemotherapy-only groups**

|  | **Chemo-Only (n=55)** | **Haplo (n=59)** | **P** |
| --- | --- | --- | --- |
| **Mean age n (range), years** | **27 (18-39)** | **25 (18-39)** | **0.210** |
| **18-29 years n(%)** | **39 (70.9)** | **46 (79.6)** |  |
| **30-39 years** | **16 (29.1)** | **13 (20.4)** |  |
| **Sex n(%)** |  |  | **0.247** |
| **Male** | **33 (60.0)** | **42 (71.2)** |  |
| **Female** | **22 (40.0)** | **17 (28.8)** |  |
| **Mean WBC n (range) *10^9^/L** | **8.1 (0.7-54)** | **8.0 (0.1-79)** | **0.412** |
| **<10 n(%)** | **32 (58.2)** | **37 (62.7)** |  |
| **10-29** | **13 (23.6)** | **12 (20.3)** |  |
| **>30** | **10 (18.2)** | **10 (17.0)** |  |
| **Diagnosis n(%)** |  |  | **0.715** |
| **B-ALL** | **42 (82.5)** | **41 (63.3)** |  |
| **T-ALL** | **13(17.5)** | **18 (36.7)** |  |
| **IKZF-1 n(%) in B-ALL** |  |  | **0.933** |
| **Positive** | **9 (21.4)** | **10 (24.4)** |  |
| **Negative** | **33 (78.6)** | **31 (75.6)** |  |
| **Induction to CR n(%)** |  |  |  |
| **CODP** | **10 (18.2)** | **7 (11.9)** | **0.346** |
| **CODP-L** | **45 (81.8)** | **52 (88.1)** |  |
| **Time to CR days(range)** | **32 (14-76)** | **34 (14-97)** | **0.113** |
| **One Course to CR1 n(%)** | **50 (90.9)** | **52(88.1)** | **0.764** |
| **MRD positive n (%)** |  |  |  |
| **Post Induction** | **21(38.2)** | **35 (59.3)** | **0.025*** |
| **Post Con-1** | **21 (38.2)** | **24 (40.7)** | **0.941** |
| **Post Con-2** | **6 (11.5)** | **11 (18.6)** | **0.170** |
| **One Course to CR1 and**  **Negative Con-1 MRD n(%)** | **29****(52.7)** | **30(50.8)** | **0.854** |
| **Centers n(%)** |  |  | **0.011*** |
| **Peking University** | **34 (61.8)** | **49 (83.1)** |  |
| **Others** | **21 (38.2)** | **10 (16.9)** |  |
| **Events n(%)** |  |  |  |
| **Relapse** | **33(60.0)** | **8 (13.6)** |  |
| **NRM** | **1 (1.8)** | **7 (11.9)** |  |

**Data are number of patients unless otherwise note**

**Table S2 Patient characteristics of the haplo-SCT and chemotherapy groups in landmark analysis**

|  | **Chemotherapy**  **at landmark**  **(n=50)** | **Haplo-SCT**  **at Landmark**  **(n=49)** | **P** |
| --- | --- | --- | --- |
| **Mean age n (range), years** | **28 (18-39)** | **22 (18-39)** | **0.052** |
| **18-29 years n(%)** | **34 (68.0)** | **37 (75.5)** |  |
| **30-39 years** | **16 (32.0)** | **12 (24.5)** |  |
| **Sex n(%)** |  |  | **0.331** |
| **Male** | **30 (60.0)** | **44 (69.4)** |  |
| **Female** | **20 (40.0)** | **15 (30.6)** |  |
| **Mean WBC n (range) *10^9^/L** | **7.5 (0.1-54.2)** | **8 (0.6-79)** | **0.864** |
| **<10 n(%)** | **29 (58.0)** | **31 (63.3)** |  |
| **10-29** | **18 (36.0)** | **13 (26.5)** |  |
| **>30** | **3 (6.0)** | **5 (10.2)** |  |
| **Diagnosis n(%)** |  |  | **0.952** |
| **B-ALL** | **37 (74.0)** | **36 (73.5)** |  |
| **T-ALL** | **13 (26.0)** | **13(26.5)** |  |
| **IKZF-1 n(%) in B-ALL** |  |  | **0.965** |
| **Positive** | **8 (21.6)** | **8 (22.2)** |  |
| **Negative** | **29 (78.4)** | **28 (77.8)** |  |
| **Induction to CR n(%)** |  |  |  |
| **CODP** | **8 (16.0)** | **6(12.2)** | **0.594** |
| **CODP-L** | **42 (84.0)** | **43 (87.8)** |  |
| **Time to CR days(range)** | **32 (14-86)** | **34 (10-97)** | **0.270** |
| **MRD positive n (%)** |  |  |  |
| **Post Induction** | **20 (40.0)** | **29 (59.2)** | **0.058** |
| **Post Con-1** | **15 (30.0)** | **14 (28.6)** | **0.877** |
| **Post Con-2** | **3 (6.0)** | **10 (20.4)** | **0.035*** |
| **Centers n(%)** |  |  | **0.056** |
| **Peking University** | **31 (55.0)** | **39 (79.6)** |  |
| **Others** | **19 (45.0)** | **10(20.4)** |  |
| **Events n(%)** |  |  |  |
| **Relapse** | **20(40.0)** | **7 (14.3)** |  |
| **NRM** | **1 (2.0)^#^** | **4 (8.2)** |  |

*** Statistical Significance**

**^#^  NRM before SCT**

**Data are number of patients unless otherwise note**

**Table S3**  **PH test in time dependent COX model**

|  | **T_COV** |
| --- | --- |
| **CR to HSCT time** | **0.0350 *** |
| **Consolidation Treatment** | **0.0500 *** |
| **Center** | **0.2213** |
| **SEX** | **0.6089** |
| **Age** | **0.9455** |
| **Diagnosis** | **0.7302** |
| **WBC** | **0.6179** |
| **IKZF1** | **0.9056** |
| **Induction Regimen** | **0.5539** |
| **Induction Days** | **0.8422** |
| **Induction MRD** | **0.8856** |
| **Con1 MRD** | **0.1387** |
| **Con2 MRD** | **0.1137** |

*** Statistical Significance**

**Table S4** **Univariate analysis of time dependent analysis**

| **Parameter** |  | **LFS** |  |  | **OS** |  |  | **CIR** |  |  | **NRM** |  |
| --- | --- | --- | --- | --- | --- | --- | --- | --- | --- | --- | --- | --- |
|  | **HR** | **95%** | **p** | **HR** | **95%** | **p** | **HR** | **0.950** | **p** | **HR** | **95%** | **p** |
| **T_COV**  **Haplo vs Chemo** | **0.434** | **0.228-0.827** | **0.011 *** | **0.314** | **0.146-0.676** | **0.003*** | **0.249** | **0.111-0.560** | **0.001*** | **6.570** | **0.802-53.802** | **0.079** |
| **Center**  **Other vs Peking** |  |  | **0.654** |  |  | **0.507** |  |  | **0.530** |  |  | **0.835** |
| **Sex**  **Female vs Male** |  |  | **0.769** |  |  | **0.350** |  |  | **0.594** |  |  | **0.557** |
| **Age** |  |  | **0.927** |  |  | **0.973** |  |  | **0.689** |  |  | **0.652** |
| **Diagnosis T vs B** |  |  | **0.131** | **2.330** | **1.156-4.696** | **0.018 *** |  |  | **0.934** | **10.591** | **2.123-52.838** | **0.004*** |
| **WBC*^$^*** |  |  | **0.107** |  |  | **0.113** |  |  | **0.577** | **1.561** | **1.122-2.172** | **0.008 *** |
| **IKZF + vs -** |  |  | **0.892** |  |  | **0.976** |  |  | **0.658** |  |  | **0.411** |
| **Induction L + vs -** |  |  | **0.418** |  |  | **0.277** |  |  | **0.488** |  |  | **0.883** |
| **CR time** |  |  | **0.956** |  |  | **0.837** |  |  | **0.545** |  |  | **0.137** |
| **CR Course 2 vs. 1** |  |  | **0.448** |  |  | **0.460** |  |  | **0.718** |  |  | **0.200** |
| **MRD post induction** |  |  | **0.847** |  |  | **0.834** |  |  | **0.265** |  |  | **0.376** |
| **MRD post Con-1** | **1.885** | **1.076-3.303** | **0.027*** |  |  | **0.295** | **2.177** | **1.174-4.035** | **0.014*** |  |  | **0.417** |
| **MRD post Con-2** |  |  | **0.112** |  |  | **0.933** | **2.034** | **0.996-4.153** | **0.051** |  |  | **0.445** |

**** Statistical Significance $ Linear with estimates of HRs for ten years difference***

**CIR:** treatment option (haplo-SCT vs chemotherapy; HR 0.249, 95% CI 0.111-0.560, P=0.001) and Con1 FCM MRD (+ vs -; HR 2.177, 95% CI 1.174-4.035, P=0.014) were risk factors that affected CIR, whereas the other variables, such as age, sex, WBC count at diagnosis, T cell or B cell, induction regimens, induction time and enrolled centers, did not influence the risk of CIR.

**NRM:** diagnosis (T vs B; HR 10.591, 95% CI 2.123-52.838, P=0.004) and WBC count at diagnosis (linear with estimates of HRs for 10*109/L. [HR] 1.561, 95% CI 1.122-2.172, P=0.008) affected NRM.

**LFS:** treatment option (haplo-SCT vs chemotherapy; [HR] 0.434, 95% CI 0.228-0.827; P=0.011) and Con-1 MRD status (+ vs -, [HR] 1.885, 95% CI 1.076-3.303, P=0.027) were potential risk factors that affected LFS. Other variables did not influence LFS.

**OS:** treatment option (haplo-SCT vs chemotherapy; HR 0.314, 95% CI 0.146-0.676, P=0.003) and diagnosis (T vs B; HR 2.330, 95% CI 1.156-4.696, P=0.018) affected OS; however, the other variables did not influence OS.

**Table S5. Multivariate analyses of CIR, NRM, LFS and OS**

| **Parameter** |  | **Crude** |  |  |  | **PS-Adjusted** |  |
| --- | --- | --- | --- | --- | --- | --- | --- |
|  | **HR** | **95%CI** | ***P*** |  | **HR** | **95%CI** | ***P*** |
| **CIR** |  |  |  |  |  |  |  |
| **Haplo-SCT vs Chemo** | **0.238** | **0.105-0.537** | **<0.001*** |  | **0.195** | **0.076-0.499** | **0.001*** |
| **Con-1 MRD + vs -** | **2.292** | **1.235-4.255** | **0.009*** |  | **3.609** | **1.562-8.340** | **0.006*** |
| **NRM** |  |  |  |  |  |  |  |
| **Diagnosis T vs B** | **9.124** | **1.820-45.74** | **0.017*** |  | **1.372** | **0.123-15.299** | **0.797** |
| **LFS** |  |  |  |  |  |  |  |
| **Haplo-SCT vs Chemo** | **0.382** | **0.199-0.735** | **0.004*** |  | **0.297** | **0.131-0.675** | **0.003*** |
| **Con-1 MRD + vs -** | **2.033** | **1.155-3.576** | **0.014*** |  | **2.825** | **1.298-6.152** | **0.009*** |
| **OS** |  |  |  |  |  |  |  |
| **Haplo-SCT vs Chemo** | **0.307** | **0.141-0.668** | **0.003 *** |  | **0.346** | **0.140-0.853** | **0.011*** |
| **Diagnosis T vs B** | **2.701** | **1.332-1.332** | **0.006 *** |  | **2.564** | **1.361-4.823** | **0.014*** |

**** Statistical Significance***
