# Supplementary material for: Comparison of haplo-SCT and chemotherapy for young adults with standard-risk Ph-negative acute lymphoblastic leukemia in CR1
Source: J Hematol Oncol. 2020 May 15;13:52. doi: 10.1186/s13045-020-00879-1 (PMC7227076; doi:10.1186/s13045-020-00879-1)
Supplement: Supplementary file 3 — Additional file 3:. Supplementary methods. [file 13045_2020_879_MOESM3_ESM.docx]

**Methods**

**Patients**

Newly diagnosed Ph-ALL YA patients were consecutively enrolled in 6 centers in China, namely, Peking University People’s Hospital, Peking Union Hospital, Wuhan Union Hospital, General Hospital of PLA, First Hospital of Soochow University and Peking University Aerospace Center Hospital, between July 2014 and Feb 2018, according to the following inclusion criteria: (1) age 18 to 39 years; (2) without high-risk features, which consisted of elevated WBC count (>30 *10^9^/L for B cell lineage or >100 *10^9^/L for T cell lineage) or high-risk cytogenetic abnormalities, such as hypodiploidy, t(v;11q23), and a complex karyotype (≥5 chromosome abnormalities) determined according to the NCCN 2013 guidelines; and (3) achieved CR after 1 to 2 cycles of induction.

Donor searches were initiated during induction. After cycles of consolidation, patients were excluded from the study if they did not meet the following criteria: remained in CR1, physical score (ECOG) 0-2 and hematopoietic cell transplantation specific comorbidity index (HCT-CI) 0-2. After two cycles of consolidation, the first choices was MSD-SCT or MUD with available donor. Subjects without suitable MSD or MUD were randomized at 1:1 to receive haplo-SCT or chemotherapy.

After 1 year of enrollment, randomization was terminated because of the patients’ intention to crossover between the two intervention groups (total enrolled n=16). The sample size was recalculated as cohort study in July 2015, and the ethics committee agreed to modify the randomization scheme to intention-to-therapy (ITT). Patients who met the recruitment criteria after the second consolidation participated fully in discussions with their doctors and then made decisions on their own regarding their intention to receive haplo-SCT. For each patient, informed consent was obtained from patients in accordance with the Declaration of Helsinki. The trial was approved by the ethics committees at all participating hospitals. Figure S1 provides an overview of the enrolled patients based on ITT.

**Diagnostics and assessment of the treatment response**

ALL was diagnosed according to the WHO 2008 criteria and as described previously. CR was defined as 5% bone marrow (BM) blasts, the absence of extramedullary disease. Minimal residual disease (MRD) detected by eight-color flow cytometry (FCM) defined as previously reported (in brief >0.01% leukemia-associated immunophenotypes (LAIP) positive cells, minimum 750,000 collected events)[1].

**Treatment protocols**

Treatment involved 1-2 cycles of induction with VCDP (intravenous vindesine 4 mg/d, days 1, 8, 15, and 22; cyclophosphamide, Cy 750 mg/m^2^/d, days 1; daunorubicin 45 mg/m^2^/d, days 1 to 3 and days 15 to 17; dexamethasone equivalent to prednisone 1 mg/kg/d, days 1 to 21) plus subcutaneous L-asparaginase 2,000 U/m^2^ once, days 19 to 28) or not. If CR occurred, patients received 2 cycles of consolidation with hyper-CVAD-B regimen (intravenous high dose of methotrexate 1 g/m^2^ d1 and cytarabine 2-3 g/m^2^, q12 h, d2-3) and a hyper-CVAD-A regimen (intravenous hyperfractionated Cy 300 mg/m^2^ q12 h, d1-3; vindesine 4mg/d, d4, d11, IV; epirubicin 60mg/m^2^, d4; dexamethasone 40 mg/d, d1-4, d11-14). Patients in the chemotherapy group received an additional 6 cycles of hyper-CVAD-B/A[2] and maintenance therapy up to 2 years (oral methotrexate and mercaptopurine). Intrathecal chemotherapy with methotrexate, cytarabine, and dexamethasone was given to all patients for at least six doses during induction and consolidation chemotherapy (before HSCT) as previously reported[3, 4]. No patient received antigen receptor (CAR) T cells (Car-T) or antibody-based therapies before the first relapse.

All SCT patients received grafts with both G-CSF mobilized bone marrow and peripheral blood. The target mononuclear cell count was 6-8×10^8^/kg of the recipient’s weight. Considering the relatively lower CIR of SR ALL CR1 patients compared with HR ALL patients, busulfan (Bu)/Cy-based conditioning rather than total body irradiation (TBI) was chosen in the current study[5]. The conditioning of haplo-SCT was: intravenous cytarabine (4 g/m^2^ per day) on days -10 to -9, intravenous Bu (3.2 mg/kg per day) on days -8 to -6, intravenous Cy (1.8 g/m^2^ per day) on days -5 to -4, oral methyl chloride hexamethylene urea nitrate (Me-CCNU; 250 mg/m^2^ per day) on day -3, and intravenous ATG (2.5 mg/kg per day; Sang Stat, Lyon, France) on days -5 to -2. The graft-versus-host disease (GVHD) prophylaxis regimen consisted of pretransplant ATG and posttransplant cyclosporine A, mycophenolate mofetil, and short-term methotrexate. Patients received low-dose corticosteroid prophylaxis with based on their risk stratification[6]. Two doses of 14.5 mg/kg Cy were given on days 3 and 4 after SCT for patients who received haplo-SCT from maternal donors or collateral relatives[7].

**End points and statistical methods**

LFS was the primary end point, which was defined as the survival period with continuous CR from CR1 after induction. MRD was not considered relapse for the LFS determination. Other endpoints included OS, CIR and nonrelapse mortality (NRM) in the whole study population, as well as GRFS in the haplo-SCT cohort. Relapse was defined as a recurrence of >5% BM blasts, the reappearance of blasts in the blood, or the development of extramedullary disease. NRM was defined as death after SCT or chemotherapy without disease progression or relapse. CIR was calculated using competing risks with NRM. Acute and chronic GVHD were evaluated and graded by a single practitioner within the program[8, 9]. GRFS events were defined as grade III-IV aGVHD, systemic therapy-requiring cGVHD (extensive or NIH severe), relapse, or death in the two years post-SCT.

To exclude bias that may arise from including patients who relapsed or died too early to receive haplo-SCT in CR1, dynamic landmark analysis and time-dependent Cox proportional hazards regression were used. The dynamic landmark points were calculated between 0 and 12 months after CR1; patients in whom death, the end of follow-up, or relapse occurred before the landmark were excluded. Patients undergoing SCT before the landmark were categorized in the haplo-SCT group, whereas those undergoing SCT after the landmark were included in the chemotherapy-only group[10]. The median time to haplo-SCT was 4.8 months from the achievement of CR1, while the median time to MSD/MUD-SCT was 4.7 months from the achievement of CR1.

As only 18 patients (30.5%) received haplo-SCT before 4 months post CR1 while 9 patients (15.3%) received haplo-SCT after 6 months post CR, we chose 6 months as the fixed landmark point to demonstrate outcomes comparing haplo-SCT and chemotherapy.

Multivariable Cox regression analyses were performed with consolidation by allo-SCT as a time-dependent covariate, in which patients receiving consolidation by chemotherapy first followed by haplo-SCT were counted as at risk in the chemotherapy group from CR1 until SCT and then as at risk in the haplo-SCT group, which was conceptually similar to a Mantel-Byar analysis[11]. The variables included in the univariate analysis were patient age (linear with estimates of HRs for differences of ten years), sex (female or male), WBC count at diagnosis (linear with estimates of HRs for differences of 10×10^9^/L), T cells or B cells, induction regimens (with or without L-asparaginase), and induction time (linear with estimates of HRs for differences of 10 days). Age, WBC count at diagnosis and induction time were calculated as continuous variables.

Other statistical methods, including comparisons of patient characteristics between two groups, Kaplan-Meier method, competitive risk model, test for proportional hazards (PH) assumption and propensity score (PS) weighting, were calculated as previously reported [12]. SPSS 24.0, R software 3.5.3 (with library “dynpred”, etc.), and GraphPad Prism 8.0 were used for data analyses and graphing.

**References:**

1. Zhao XS, Liu YR, Xu LP, Wang Y, Zhang XH, Chen H, Chen YH, Han W, Sun YQ, Yan CH, et al: **Minimal residual disease status determined by multiparametric flow cytometry pretransplantation predicts the outcome of patients with ALL receiving unmanipulated haploidentical allografts.** *Am J Hematol* 2019, **94:**512-521.

2. Kantarjian HM, O'Brien S, Smith TL, Cortes J, Giles FJ, Beran M, Pierce S, Huh Y, Andreeff M, Koller C, et al: **Results of treatment with hyper-CVAD, a dose-intensive regimen, in adult acute lymphocytic leukemia.** *J Clin Oncol* 2000, **18:**547-561.

3. Wang Y, Liu QF, Xu LP, Liu KY, Zhang XH, Ma X, Wu MQ, Wu DP, Huang XJ: **Haploidentical versus Matched-Sibling Transplant in Adults with Philadelphia-Negative High-Risk Acute Lymphoblastic Leukemia: A Biologically Phase III Randomized Study.** *Clin Cancer Res* 2016, **22:**3467-3476.

4. Han LJ, Wang Y, Fan ZP, Huang F, Zhou J, Fu YW, Qu H, Xuan L, Xu N, Ye JY, et al: **Haploidentical transplantation compared with matched sibling and unrelated donor transplantation for adults with standard-risk acute lymphoblastic leukaemia in first complete remission.** *Br J Haematol* 2017, **179:**120-130.

5. Giebel S, Marks DI, Boissel N, Baron F, Chiaretti S, Ciceri F, Cornelissen JJ, Doubek M, Esteve J, Fielding A, et al: **Hematopoietic stem cell transplantation for adults with Philadelphia chromosome-negative acute lymphoblastic leukemia in first remission: a position statement of the European Working Group for Adult Acute Lymphoblastic Leukemia (EWALL) and the Acute Leukemia Working Party of the European Society for Blood and Marrow Transplantation (EBMT).** *Bone Marrow Transplant* 2018.

6. Chang YJ, Xu LP, Wang Y, Zhang XH, Chen H, Chen YH, Wang FR, Han W, Sun YQ, Yan CH, et al: **Controlled, Randomized, Open-Label Trial of Risk-Stratified Corticosteroid Prevention of Acute Graft-Versus-Host Disease After Haploidentical Transplantation.** *J Clin Oncol* 2016, **34:**1855-1863.

7. Wang Y, Chang Y, Chen L, Xu L, Bian Z, Zhang X, Yan C, Liu K, Huang X: **Low-dose post-transplant cyclophosphamide can mitigate GVHD and enhance the G-CSF/ATG induced GVHD protective activity and improve haploidentical transplant outcomes.** *Oncoimmunology* 2017, **6:**e1356152.

8. Przepiorka D, Weisdorf D, Martin P, Klingemann HG, Beatty P, Hows J, Thomas ED: **1994 Consensus Conference on Acute GVHD Grading.** *Bone Marrow Transplant* 1995, **15:**825-828.

9. Jagasia MH, Greinix HT, Arora M, Williams KM, Wolff D, Cowen EW, Palmer J, Weisdorf D, Treister NS, Cheng GS, et al: **National Institutes of Health Consensus Development Project on Criteria for Clinical Trials in Chronic Graft-versus-Host Disease: I. The 2014 Diagnosis and Staging Working Group report.** *Biol Blood Marrow Transplant* 2015, **21:**389-401 e381.

10. Nicolaie MA, van Houwelingen JC, de Witte TM, Putter H: **Dynamic prediction by landmarking in competing risks.** *Stat Med* 2013, **32:**2031-2047.

11. Delgado J, Pereira A, Villamor N, Lopez-Guillermo A, Rozman C: **Survival analysis in hematologic malignancies: recommendations for clinicians.** *Haematologica* 2014, **99:**1410-1420.

12. Lv M, Wang Y, Chang YJ, Zhang XH, Xu LP, Jiang Q, Jiang H, Lu J, Chen H, Han W, et al: **Myeloablative Haploidentical Transplantation Is Superior to Chemotherapy for Patients with Intermediate-risk Acute Myelogenous Leukemia in First Complete Remission.** *Clin Cancer Res* 2019, **25:**1737-1748.
